# How relevant is environmental quality to per capita health expenditures? Empirical evidence from panel of developing countries

**DOI:** 10.1186/s40064-016-2505-x

**Published:** 2016-06-29

**Authors:** Adamu Yahaya, Norashidah Mohamed Nor, Muzafar Shah Habibullah, Judhiana Abd. Ghani, Zaleha Mohd Noor

**Affiliations:** Economics Department, Faculty of Economics and Management, University Putra Malaysia, Selangor, Malaysia

**Keywords:** Environmental quality, Health expenditure, Panel cointegration

## Abstract

**Background:**

Developing countries have witnessed economic growth as their GDP keeps increasing steadily over the years. The growth led to higher energy consumption which eventually leads to increase in air pollutions that pose a danger to human health. People’s healthcare demand, in turn, increase due to the changes in the socioeconomic life and improvement in the health technology. This study is an attempt to investigate the impact of environmental quality on per capital health expenditure in 125 developing countries within a panel cointegration framework from 1995 to 2012.

**Results:**

We found out that a long-run relationship exists between per capita health expenditure and all explanatory variables as they were panel cointegrated. The explanatory variables were found to be statistically significant in explaining the per capita health expenditure. The result further revealed that CO_2_ has the highest explanatory power on the per capita health expenditure. The impact of the explanatory power of the variables is greater in the long-run compared to the short-run. Based on this result, we conclude that environmental quality is a powerful determinant of health expenditure in developing countries.

**Conclusion:**

Therefore, developing countries should as a matter of health care policy give provision of healthy air a priority via effective policy implementation on environmental management and control measures to lessen the pressure on health care expenditure. Moreover more environmental proxies with alternative methods should be considered in the future research.

## Background

The upturn in the economic activities in most developing countries goes along with an increase in energy consumption which eventually leads to increase in air pollutions that pose danger to human health. People’s healthcare demand, in turn, increase due to the changes in the socioeconomic life and improvement in the health technology. The financial implication of outdoor air pollution in developing countries has been marked to the tune of 5 % of their GDP (United Nations Environment Program 2016). Frogner (2008) in his proposition argue that the growth rate of health care expenditure is quite alarming because it is greater than the rate at which GDP grows. This trend provoke investigations on the health expenditure determinants.

There is a substantial growth of studies regarding the determining factors of healthcare expenditure which may not be unconnected with the persistence increase in the healthcare expenditure over the years. Gerdtham et al. (1992), Gbesemete and Gerdtham (1992), Murthy and Ukpolo (1994, 1995), Hansen and King (1996), Matteo and Matteo (1998), Gerdtham and Lothgren (2000), Murthy and Okunade (2000), Herwartz and Theilen (2003) have examined a number of determinants of health expenditure comprising of both economic and non-economic factors such as ageing, population, income, amount of doctors, rate of women labor force participation, government health care finance, external aid, urbanization etc. What is still new in the existing research is the role of air quality on the growth of healthcare expenditure. The novelty of this study is the investigation particularly on the impact of air pollutants such as; carbon monoxide emissions, sulfur, nitrous and carbon dioxide emissions on per capita health spending in developing countries.

The term environment is a multifaceted concept that comprises of land, air, water and our choice of air pollution to represent its quality is deliberate since it has been established that air pollution is the main environmental risk to health (WHO 2003). Improvement in the air quality is likely to decrease global burden of disease from various respiratory ailments. This may consequently lead to a reduction in both individual and aggregate health budgets.

Discharging contaminated gasses with chemicals into the air is one of the major roots of air pollution. Developing countries become more vulnerable to the pollution impacts as they attract hefty polluting industries. This is either as a result of the cheap, abundant labour supply and inputs or weak environmental regulation compared to the developed countries. The heavy polluting industries constitute the major outlet of the air contaminants (USEPA 2010). Vidal and Adam (2007) argue that health effect of the industrial emissions are more dangerous than vehicle discharges.

Air quality can represent the state of the air around us which can be regarded as clean if it is clear and uncontaminated. Uncontaminated air is necessary to maintain the subtle balance of life on this earth not just for human beings but for animals, vegetation, water and soil as well. Basic sources of poor air quality include; emissions from both natural and artificial environment. The air pollutants may either be outdoor (Ambient) or indoors. The former denotes to the quality of outside air in our surrounding environment. It is normally measured near ground level, away from direct sources of pollution. The latter refers to the air in enclosed spaces like home, schools or workplaces which can also be contaminated with pollutants that have leaked in from the outside. The quality of the air is termed poor when contaminants reach high enough concentrations to endanger human health and the environment (British Columbia air quality 2016). Sulfur dioxide (SO_2_) major source is the fuel combustion from vehicles and other engines. It responds easily with other substances in forming harmful compounds, like sulfurous acid, sulfuric acid, and sulfate particles. It causes chest and an asthmatic problem for human through the breathing of the substance (Air quality fact sheet 2005). Nitrous oxide (NOX) is one of the Earth’s nitrogen cycle in the atmosphere which emanates from human activities such as industrial processes, agricultural activity, waste management, and fossil fuel combustion. This compound is largely formed from agricultural activities (US EPA, 2015). Carbon monoxide (CO) is formed from the partial burning of natural gas and other carbon material like coal, gasoline propane, kerosene etc. it harms human because it moves oxygen in the blood and attacks the heart and other important organs of oxygen. By implication, the deterioration of the air quality causes various sicknesses which affect both public and individual health budgets.

Sources of the air pollutants in developing countries are not different with what is obtainable in the developed world. The only difference is the control standards through viable enforceable environmental policies, laws and acts. The stringent environmental laws in the developed world are responsible for turning the developing countries into pollution heavens for highly polluting industries.

**Fig. 1:**
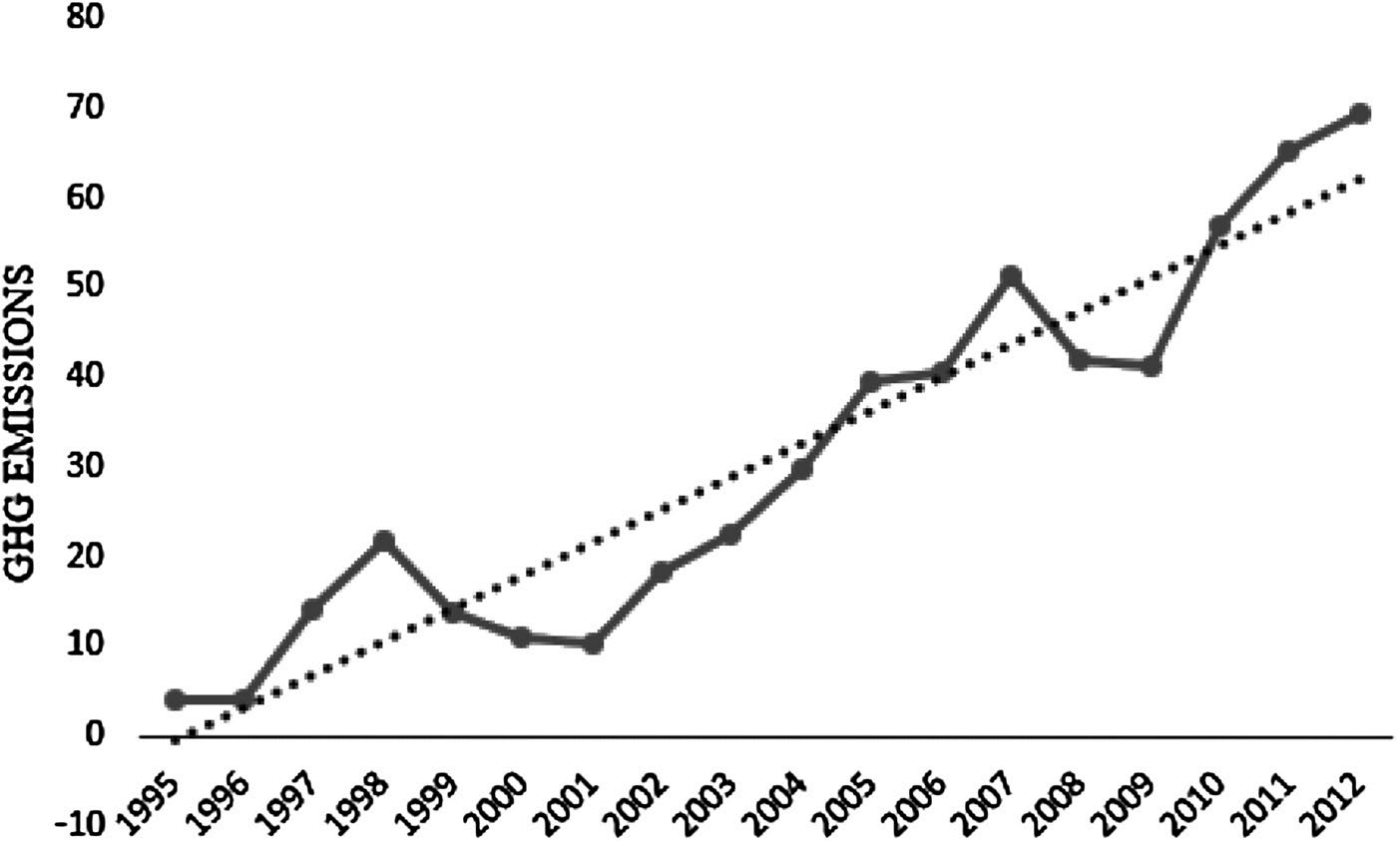
Total greenhouse gas emissions in Developing Countries (% change from 1990). *Data source* WDI World Bank Database 2015

 Greenhouse gases indicated in the Fig. 1 has shown an up trending movement from 1995 to 2012. This trend has some effect on health budgets since it has a direct link with human health. WHO report in 2012 proved that three million seven hundred thousand deaths was attributed to ambient (outdoor) air pollution. Nearly 88 % of those deaths happened in low- and middle-income countries and the highest burden was recorded in the Western Pacific and South East Asian. In the same year, 4.3 million deaths were caused by household (indoor) air pollution. Virtually all these deaths occurred in developing countries. In total, air pollution is accountable for almost 1 in every 8 deaths (World Health Organization 2014).

**Fig. 2:**
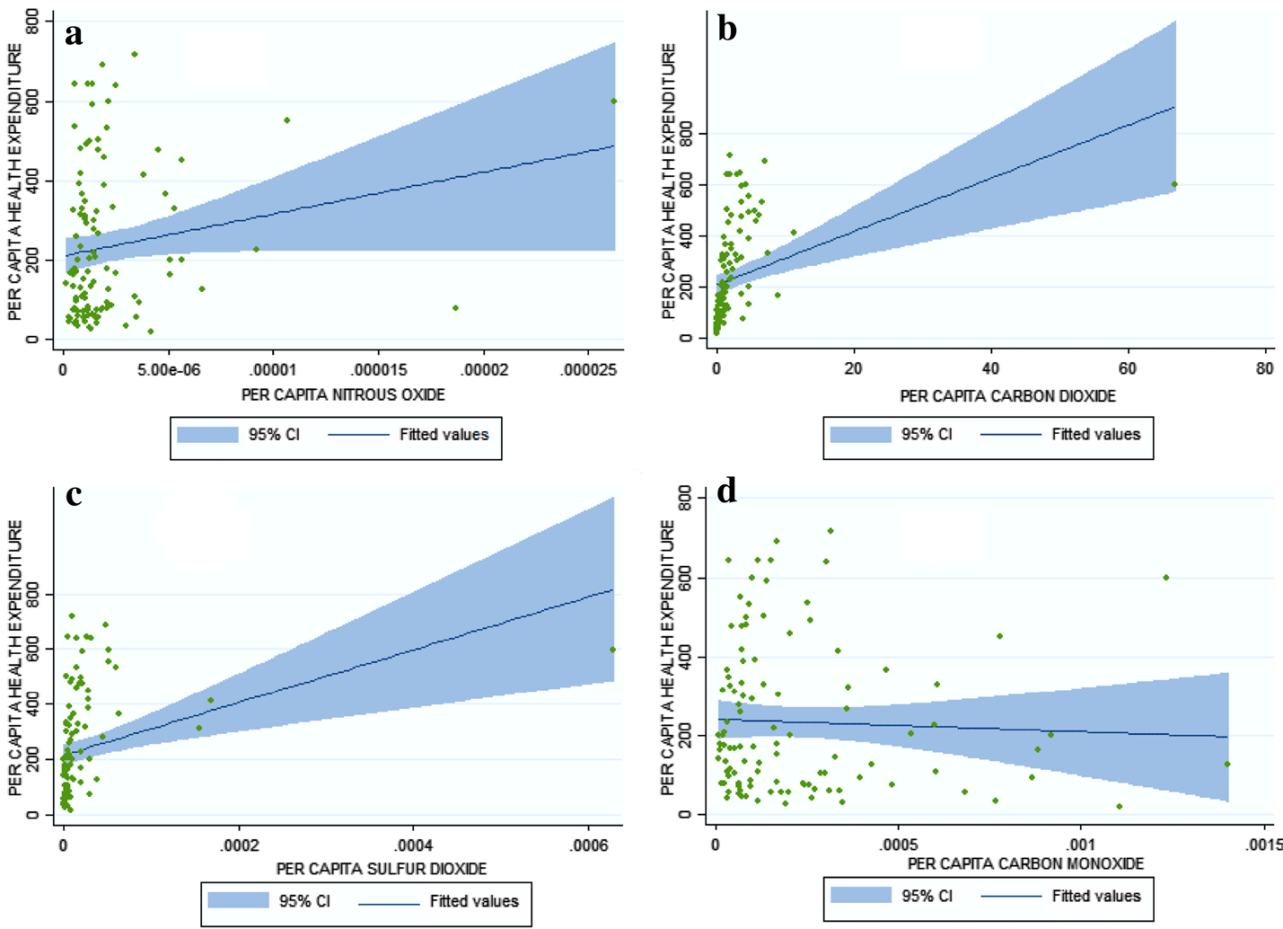
Scatter plots for per capita health expenditure and NOX (**a**); CO2 (**b**); SO2 (**c**); CO (**d**) in developing countries

Figure 2a, b represent scatter plots for per capita nitrous oxide (NOX), per capita carbon dioxide (CO_2_) and real per capita health expenditure in developing countries. Both plots have maintained positive link between health expenditure and the emissions. The relationship between sulfur dioxide per capita (SO_2_) and percapita health expenditure appeared positive but negative is the relationship shown with per capita carbon monoxide (CO). it indicates that as the CO increases, per capita health expenditure decreases (Fig. 2c, d). On the contrary, the empirical result from OECD countries in Narayan and Narayan (2008) showed a positive link between carbon monoxide (CO) and health expenditure. Furthermore, Boden et al. (2010) opined that the global fossil fuels carbon emissions from 1900 to 2008 have remarkably increased by over 16 times. Developing countries though have less polluting capacity compared to the developed yet they have the highest burden of diseases. Globally, 80 % of all kinds of diseases and ¼ of the global burden of the disease are caused by environmental vulnerability (World Health Organization 2011). This culminates to the higher risk because of poverty, lack economic capacity to invest in technology, poor environmental legislation and control measures (Briggs 2003).

Pearce ad Turner (1991) argued that the expenses of environmental pollution are unarguable and become a burden on government finances as it leads to increased demand for health care expenditures. The connection among environmental conditions, public policy on the public health system, prices of health care bond on fee repression in the health care sector (Jarrett et al. 2003). Air pollution is the main source of environmental destruction which led to the health suffering in the society as it negatively affects human health and labour productivity. Consequently industrial output and by large national output will be subdued and the entire economy will be affected.

The relationship between air pollution and human health has been established in the literature. For example, Hausmann et al.(1984), Ostro (1987), Ostro and Rothchild (1989), and Zuidema and Nentjes (1997), have studied the relationship between total suspended/fine particles and lost work days as a result of sickness as Hansen and Selte (2000) investigates on leave and labour output, generally they conclude that the relationship between air pollution and human health is inverse.

Narayan and Narayan (2008), Assadzadeh et al. (2014) make attempt to examine the impact of the environmental quality in determining the amount of expenditure on health. Our work defers with theirs both in scope and the choice of environmental proxy. Narayan and Narayan applied panel cointegration on eight OECD countries without CO_2_ while the later study eight petroleum exporting countries considering only CO_2_ as the proxy for the environmental quality. Our study considered panel cointegration of 125 developing countries owing to the persistent health complications as well as the steady increase in health expenditures in developing countries. Another parting point is our consideration of broader air emissions in addition to the environmental quality proxies they used.

Pedroni (1999), Pedroni (2000), and Westerlund (2006) put it as conditions that the fundamental variables in our models need to be integrated of order one and if this is satisfied, then, we conduct panel cointegration. These conclusions have consequences for prerogative on elasticities because elasticity can be claimed to be a long run only when the proxies are non-stationary and collectively they exhibit a long-time relationship. Moreover, if a long-time association happens, it overlay the way for evaluating short-time responses through variables transformation into first differences. Furthermore, if long time association is not established at that point we can only evaluate short time elasticities. This is an essential stage since irrespective of the elasticity is long-time or short-time is imperative for planning resolutions plus further econometric modelling, like future projecting.

Examining the determining factors of health spending in developing countries is the main aim of this study based on some novelties; first, application of panel cointegration framework in measuring the effect of air quality on health expenditure. The model we used is the modified bivariate model that comprises only income and health expenditure by Newhouse (1977) to a multivariate model involving; per capita health expenditure depending on CO_2_, nitrous, and sulfur oxide emissions representing environmental quality. Secondly, rigor panel data analysis of health spending determinants for developing countries was carried out using a panel cointegration recommended by Pedroni (1999) by estimating long-run elasticities. Similarly, we equally determine short run elasticities from long run to spot out the difference in response over time.

This paper is structured into six sections; “Introduction”, Review of the literature on determinants of health”, “Model specification”, “Discussion of techniques used”, “Discussion of results and finally”, “Presents conclusion with policy recommendations” section.

## Literature reviewed

As pointed out previously, there are many researches on the factors determining expenditures on health care but then it is very difficult to analyze all of the literature. For this reason, our work reviewed works that are relevant to the subject matter under study. Jerrett et al. (2003) used cross-sectional statistics for forty-nine countries in Ontario Canada to examine the link between health expenditures and the quality of environment represented by air pollutants and government spending for protecting the environment. On one hand, they argued that countries with more pollutant emissions were found to have higher per head expenditure on health and those that spent enough for environmental protection have lesser expenditures on health. Air pollution has a direct and statistically significant impact on child hospitalization as a result of Asthma (Neidell 2004).

A study in the USA between 1960 and 1987 on per capita health expenditure and its determining factors revealed that health services and Medicare prices, GDP per capita, age, the number of physicians, ratio of public health expenditures were found to be cointegrated and important determinant for the endogenous (Murthy and Ukpolo 1995).

It has been argued using a logit model from Oslo that, the number of sick leave increase with an increase in the amount of particulate matter from the air. Human health deteriorates as the air gets contaminated, labour productivity reduces as a result of more sick leaves and ultimately the amount of trade and industry deals drops (Hansen and Selte 2000).

Karatzas (2000) openly examined in the USA, the link amongst economic and per head health spending, population structure and health stock and his conclusions were; income distribution, income per head, nurses and doctors had a direct effect on per head health spending significantly. On the other hand, hospital beds, health price index, towns with over 100,000 people had an inverse relationship with per head health spending significantly.

Income, as well as age, has a direct effect on per head health expenditures as federal transfers have an indirect effect on per head health expenditures from 1965 to 1991 in Canada as examined by Matteo and Matteo (1998).

Gerdtham et al. (1992) examined 19 OECD countries using a cross-sectional data, the relationship between per head health expenditure, income per head, amount of doctors, women labour force, the percentage of people living in cities and populace above 65 birth years in 1987. Their discovery was that income and populace above 65 years showed a direct substantial effect on per head health spending but urbanization and doctors had an indirect effect on per head health spending significantly. Using panel data, twenty OECD countries studied by Hitiris and Posnett (1992) confirmed that from 1960 to 1987, the population in above 65 years category and income both had a positive and statistically significant effect on health expenditures.

## Data and methods

In consideration of 125 developing countries^1^ in this study, data availability determines the size of the countries involved. Annual data from 1995 to 2012 was used. All variables used in this study were from two sources. These sources include World Bank database and EDGAR 4.2 (2011). The data from EDGER are not available beyond 2008 hence considering their disposition the remaining few years values for a single variable were extrapolated. Although there is a divergence of opinion on its reliability yet it is consistent and objective following Armstrong’s (2001) argument.

### Model specification

As stated in the introduction, our model is the extension of the bivariate model of Newhouse (1977) where health expenditure was specified as a function of income. Most if not all models of the determinants of health expenditures were rooted from Newhouse’s model. Notably, this model has been expanded by studies such as in Karatzas (2000) where health expenditure had been expressed as a function of several socioeconomic factors. Multivariate model framework emerged as a result of these modifications.

Our novelty in this regard is the expression of per head health expenditure as a function of environmental quality using four proxies for the environment in a multivariate framework. Our study differs with Narayan and Narayan’s (2008) work by the scope, as they studied OECD countries, our focus is on developing countries because they bear more consequences of the air impurity(USEPA 2010). Our model formation looks thus:

1 $$lphex_{it} = \beta_{0} + \beta_{1} lY_{it} + \beta_{2} lNO{\text{x}}_{it} + \beta_{3} lSO2_{it} + \beta_{4} lCO_{it} + \beta_{5} lCO2_{it} + \varepsilon_{it}$$

phex represents health expenditures per capita in real terms (U$ PPP), Y stands for income per head in real term (US$ PPP), NOx equates nitrous dioxide, SO2 represents sulfur dioxide, CO is the carbon monoxide emissions, CO_2_ = carbon dioxide and $$\varepsilon_{it}$$ tands for the error term restricted within the classical statistical assumptions. For the sake of clarity and understanding, all variables were converted into log form to be interpreted as elasticity.

### A priori expectation

$\beta_{1} , \uparrow \left[ {\beta_{2} + \beta_{3} + \beta_{4} + \beta_{4} } \right] > 0.$ t is expected that rise in income and decay in the quality of environment via an increase in emissions to have a positive impact on health expenditures. The more a country’s income grows the more they spend on their health. Studies that empirically supported this premise include Gerdtham et al. (1992), Murthy and Ukpolo (1994, 1995) Hansen and King (1996). However, deterioration in the quality of environment affects human health negatively and decline in people’s health claims more expenditure on the health care.

Medical experts have empirically proven the existence of a direct relationship between air pollution and human mortalities. For example Pope et al. (1992); Schwartz (1994a); Shogren (2001); Mead and Brajer (2005) argued that air pollution is related directly to respiratory mortality. Pneumonia and acute frustrating lung diseases death are positively linked to air pollution as argued by Schwartz and Dockery (1992) and Schwartz (1994b). Air pollution is responsible for the heart-related death (Wordly et al. 1997. On a broader perspective, many kinds of literature for example Dockery et al. (1992) and Dockery et al. (1992) hold the belief that all causes of death are directly related to air pollution.

Specifically, carbon mono oxide is a toxic smoke that is formed as a result of the improper burning of carbon in the fuel. This may emanate from vehicle exhaust, stationary engines (like construction & grinding machines, manufacture of industrial metals & chemicals machines), forest fire, domestic wood and bush burning etc. Man’s health gets affected by it through the central nervous system and cardiovascular diseases. Development of acid rain and ozone are nurtured by nitrogen oxide discharges concentration of which causes obstruction in the respiratory system like asthma for both younger and elderly people. Sulfur oxide formation originates from the burning of coal and oil. Its inhalation can cause a nasal and esophagus obstruction that leads to difficulties in breathing. Moreover, as specified above, these pollutants (nitrogen dioxide, sulfur oxide, carbon monoxide) were proved to be culpable of various health complicated issues (Spix and Wichmann, 1996).

Ultimately, carbon monoxide, nitrogen oxide discharges, and sulfur oxide emissions all deteriorate environmental quality via air contamination that can, therefore, affect health negatively. Consequently, health care demand escalates.

### Technique of analysis

#### Test for panel unit root

Adopting Im et al. (2003) panel unit root test, a sample of N groups observed over a period of time T has a conventional ADF test form as;

The hypothesis of a unit root in the panel is specified as:

2 $$\Delta y_{it} = \alpha_{i} + \beta_{i} Y_{it - 1} + \sum\limits_{j = 1}^{k} {\psi_{ij} } \Delta X_{it - j} + \varepsilon_{it}$$

$$\begin{aligned}& \begin{array}{ll} {\text{Null }}&{\text{H0}}: \beta_{i} = 0,\;{\text{ for all i}} \\ {\text{Alternative }} & {\text{H1}}: \beta_{i} < 0 \, \;{\text{where i}} = 1,2, \ldots ,{\text{ N}}1 \hfill \\ \end{array} \\ & \qquad\beta_{1} = \, 0, \quad i \, = \, N1 \, + 1, \, N2 \, + 2, \, N \end{aligned}$$

Im et al. (2003) suggest LM-bar ($\overline{\text{LM}}$) statistic, which permits different serial correlation patterns through countries, as:

3 $$\overline{\text{LM}} = \frac{1}{N}\sum\limits_{j = 1}^{k} {LM_{iT} (pi,pi)}$$

For this hypothesis to be tested, Im et al. (2003) suggest a standardized LM-bar statistic in the following form:

4 $$\psi_{\overline {{\text{LM}}}} = \frac{{\sqrt {{\text{N}}\left\{ {{{\overline {{\text{LM}}} }_{{\text{NT}}}} - \frac{1}{{\text{N}}}\sum\nolimits_{{\text{j}} = 1}^{\text{N}} {{\text{E}}\left[ {{\text{L}}{{\text{M}}_{{\text{IT}}}}({{\text{p}}_{\text{i}}},0)\left| {{\beta _i} = 0} \right.} \right]} } \right\}} }}{{\sqrt {\frac{1}{{\text{N}}}\sum\nolimits_{{\text{j}} = 1}^{\text{N}} {\operatorname{var} \left[ {{\text{L}}{{\text{M}}_{IT}}({p_i})\left| {{\beta _i} = 0} \right.} \right] \Rightarrow {\text{T}},{{\text{N}}_{N(0,1)}}} } }}$$

Breitung (2000) proposed a panel unit test in this form:

5 $$y_{it} = \alpha_{it} + \sum\limits_{k = 1}^{p + 1} {\beta_{ik} } X_{t - k} + \varepsilon_{it}$$

This test is based on two assumptions that:

1. There is cross-section independence
2. Residuals are i.id., then followed by the null hypothesis which process is difference stationary as:

6 $$H0: = \mathop \sum \limits_{k = 1}^{p + 1} \beta_{ik} - 1 = 0$$

Assuming the panel series is stationary, the alternative hypothesis looks thus:

7 $$H1\text{ := } \mathop \sum \limits_{k = 1}^{p + 1} \beta_{ik} - 1 < 0\quad {\text{for\; all}} \; i$$

The test statistics has been constructed by the following transformed vector:

8 $$Y_{i}^{*} = AY_{i} = \left\{ { y_{i1}^{*} , y_{i2}^{*} , \ldots y_{iT}^{*} } \right\}^{\prime}$$

9 $$X_{i}^{*} = AX = \left\{ { x_{i1}^{*} , x_{i2}^{*} , \ldots x_{iT}^{*} } \right\}^{\prime}$$

These two lead to the following test statistic:

10 $$\lambda_{B} = \frac{{ \mathop \sum \nolimits_{i = 1}^{N} \sigma_{i}^{ - 2} Y_{i}^{*'} X_{i}^{*'} }}{{\sqrt {\mathop \sum \nolimits_{i = 1}^{N} \sigma_{i}^{ - 2} X_{i}^{*'} A 'AX_{i}^{*} } }}$$

Equation 8 portrayed a standard normal distribution.

Panel co-integration test by Pedroni starts by estimating the panel cointegration regression as follows:

11 $$lphex_{it} = \beta_{0i} + \beta_{1i} lY_{it} + \beta_{2i} lNI_{it} + \beta_{3i} lSU_{it} + \beta_{4i} lCO_{it} + \beta_{5i} lCO_{it} + \varepsilon_{it}$$

where t = 1,…, T; i = 1,…,N. In this respect, T denotes the number of observations over time and N stands for the panel individual members. After the estimation we stock the residuals $\widehat{\varepsilon }it$. Subsequently we take differentiate of the original data series for individual members and compute the residuals for the differenced model:

12 $$\Delta lphex_{i,t} = \theta_{1i} \Delta lY_{it} + \phi_{i} \Delta lNOx_{i,t} + \omega_{i} \Delta lSOx_{i,t} + \lambda_{i} \Delta lCO_{i,t} + \delta_{i} \Delta lCO2_{i,t} + \eta_{i,t}$$

Next is the computation of the long- run variance of $\widehat{\eta }$ it then using the residual *ɛ*_*it*_ of the original cointegrating equation, we calculate the autoregressive model.

Through the stages above, the following statistics: Panel v-statistics; Panel rho-statistics; Panel pp-statistics; Panel ADF-statistics; Group rho-statistics; Group pp-statistics; and Group ADF-statistics will be realized and we apply the suitable mean and variance adjustment terms as suggested in Pedroni (1999).

## Results and discussions

### Panel unit root tests

The essence of this tests is to verify the integration order of our variables owing to the major condition for panel cointegration test that all variables involved in our model must possess the key property to be non-stationary and integrated of the same order. Within the panel data framework, we used two different panel unit root tests i.e. Im et al. (2003) and Breitung (2000) to ascertain whether all variables are panel non-stationary or not. The two tests’ aims are the same but they differ in their null hypothesis formation. While IPS treat null as a unit root based on common unit root process, Breitung assumes individual unit root process.

As reported in Table 1, we discovered that both tests (IPS & Breitung) were unable to reject the null hypothesis that at level, all variables have unit root (i.e. they are panel non-stationary). On the other hand, after the first difference of the log level variables, all variables appeared to be stationary hence both tests reject the null hypothesis of the unit root at 1, 5 or 10 % respectively. These results satisfied the key condition for co-integration test.

**Table 1:** IPS and Breitung panel unit root tests (explained variable)

Variables	Order	IPS test	Breitung *t* test
Ln PHEX	Level	7.52540 (1.0000)	0.28899 (0.6137)
	1st Difference	16.7576 (0.0000)***	−7.77561 (0.0000)***

p values are in parenthesis while *** represent 1 % level of significance

### Panel co-integration test

Since both the regressand and the regressors met the condition of bieng integrated of same order 1 (at the first difference), the next is to undertake the panel co-integration test. As reported in Table 2, the Pedroni (1999) panel cointegration test result showed that per capita health expenditure and carbon mono oxide, nitrogen oxide and sulfur oxide emissions were panel cointegrated hence there is a long-run relationship among them.

**Table 2:** IPS and Breitung panel unit root tests (explanatory variables)

Variables	Order	IPS test	Breitung *t* test
ln CO	Level	1.15244 (0.1246)	0.44468 (0.6717)
	1st Difference	29.9108 (0.0000)***	−19.8485 (0.0000)***
ln SO2	Level	0.23003 (0.4090)	1.12019 (0.8687)
	1st Difference	24.4075 (0.0000)***	−15.7590 (0.0000)***
ln NOX	Level	0.85669 (0.1958)	−1.11860 (0.1317)
	1st Difference	27.5209 (0.0000)***	−21.6764 (0.0000)***
ln CO_2_	Level	1.61645 (1.0000)	−1.01383 (0.1553)
	1st Difference	1.45173 (0.0000)***	−7.19682 (0.0000)***
lnY	Level	9.33569 (1.0000)	5.43386 (1.0000)
	1st Difference	19.7009 (0.0007)***	−8.11925 (0.0000)***

p values are in parenthesis while *** represents 1 % level of significance

Panel PP statistic, panel ADF statistic, Group PP and group ADF statistic indicate panel cointegration at 1 % each. Since most of the tests are significant at 1 % level of significance (Table 3), then it is proved that our variables are panel cointegrated and our model is valid.

**Table 3:** Pedroni’s Panel cointegration test

Panel v-statistic	−1.640758 (0.9496)
Panel rho-statistic	2.973318 (0.9985)
Panel PP-statistic	−10.24536 (0.0000)***
Panel ADF-statistic	−11.96280 (0.0007)***
Group rho-statistic	8.307141(1.0000)
Group PP-statistic	−19.49143 (0.0000)***
Group ADF-statistic	−17.32064 (0.0000)***

p values are in parenthesis while *** represents 1 % level of significance

## Discussion of the panel elasticities

Two estimators were used to estimate the long-run elasticities for our specified model. The results for both long and the short-run elasticities are reported in Table 4. Starting with the long-run, the first estimator results indicate that CO, CO_2_ and Y are statistically significant at 1 % whereas, SO_2_ and NOX are significant statistically at 5 % and 10 % respectively. For the second estimator, all of the explanatory variables are significant statistically at 1 %. This implies that the variables are real determinants of health expenditure in the panel of the developing countries within the framework of our model. Theoretically, the relationship between variables is normally expressed through the direction of their movement.

**Table 4:** The long-run and the short-run results

	Long-run elasticity		Short-run elasticities		
	Panel OLS	Panel DOLS		Coefficient	t-statistics
lnCO	0.108518 (0.013020)***	0.113199 (0.035228)***	∆lnCO	0.045728*	1.783750
lnSO_2_	0.040646 (0.017187)**	0.038134 (0.072189)***	∆lnSO2	−0.020647	−0.436428
lnNOX	0.020996 (0.016080)*	0.220285 (0.052855)***	∆lnNOX	0.009313	0.244280
lnCO_2_	0.223499 (0.027769)***	0.237879 (0.128033)***	∆lnCO_2_	0.153499***	2.342642
lnY	0.679864 (0.019573)***	0.693738 (0.048045)***	∆lnY	0.278332***	2.254674
			ECT_t−1_ = −0.182937***		

p values are in parenthesis while ***, **, * represent 1, 5 & 10 % level of significance

In this analysis, the direction of the relationship as indicated by the signs of coefficients for all variables are positive. This suggests that an increase in the quantity (in percentage) of each explanatory variable will lead to a corresponding increase in the health expenditure. The impact of each variable depends on the magnitude of its coefficient. For example, the OLS estimator result shows that1 % increase in CO, SO_2_, NOX, CO_2_ and Y will lead to a corresponding increase in per capita health expenditure by 11, 4, 2, 22 and 67 % respectively. We noticed that apart from income(Y), CO_2_ has the highest explanatory power on health expenditure in the model followed by CO. The variable with the least explanatory power is NOX followed by SO_2_. The trend of the relationship exhibited in terms of signs and order of magnitude from the two estimators are almost same. Noteworthy is the robustness of our result exposed through the closeness of the coefficients and statistical significant of the variables from both estimators.

The short-run elasticities result is quite different from the long-run in many instances. First, the sign of the coefficients is not the same for all variables. Secondly, some variables are not significant statistically. We found out that all variables are statically significant except SO2. Considering the level of its significance SO2 does not require thorough interpretation in this context because it appears not relevant in our model hence it does not influence health expenditure in developing countries. Notably, the explanatory power of the variables in the short-run is comparably lesser in magnitude than in the long-run. For example, the coefficient of CO falls from 11 % in the long-run to 4.6 % in the short-run. Similarly, CO_2_ has also fallen from 22 to 15 % in the short-run. This implies that the impacts of the air quality on health expenditure increase with time since the long-run coefficients are greater than the short-run coefficients.

Another peculiarity of this result is that apart from income, CO_2_ and CO still maintained their greater impacts on health expenditure over the others. This is because even though their coefficients drops down from long-run to the short-run results, they still have higher coefficients than the rest of the explanatory variables except income.

Moreover, the coefficient of the error correction term (ECTt-1) is −0.182927 and it is statistically significant at 1 %. The negative sign of the ECT denotes the tendency of a system returning to equilibrium after a shock. We hereby conclude that since the ECT is negative and significant, health expenditure can return to the equilibrium after any shock in the system. This conclusion is based on the fact that error correction term always measures the speed of adjustment to the equilibrium path after a shock. Similarly, since ECT measures a speed of adjustments, its coefficient (0.18) is the speed rate at which the system moves back to normalcy.

To be more explicit, the total period is equal to 1/ECT = 1/0.18 = 5.5. This implies that after a shock, it takes about six (6) years to return to the equilibrium. We can, therefore, conclude that the speed of adjustment is reasonable since adjustment from macroeconomic shocks normally falls within this range of between 5 and 10 years.

## Conclusion

The general finding of this study indicates that environmental quality proxy by air pollutants have a significant direct relationship with health expenditures in developing countries. The long-run and the short-run estimators’ results further revealed that the impacts of the air pollutants on the per capita health expenditure increases over time. Similarly, the closeness of the results from the two estimators and the short-run analysis proved the robustness of our findings. Moreover, among the pollutants, CO_2_ commands the highest explanatory power on health expenditure than the other explanatory variables. Our findings did not in any way contradict the Newhouse (1972) grand theory of health expenditure since income is positive and significant throughout the analysis and its explanatory power on health expenditure is also maintained. Our aim of introducing the environmental variables into Newhouse’s model within the panel of developing countries has also been achieved. Based on these finding we are therefore concluding that the massive influx of high polluting companies into developing countries coupled with the expansion of the economic activities in developing countries degrades the environment that further exact pressure on health budgets in developing countries.

Finally our results suggest that developing countries should as a matter of health care policy give the healthy air a priority via effective policy implementation on environmental management and control measures to lessen the pressure on health care expenditure. Moreover, pollutants like particulate matter (PM), land and water pollutants proxies should be coopted for further investigations.

## Abbreviations

CO: carbon monoxide
CO_2_: carbon monoxide
GDP: gross domestic product
NOX: nitrous oxide
OECD: organization for economic cooperation and development
SO_2_: sulfur dioxide
Y: income per capita

## Footnotes

1 Albania Algeria, Angola, Armenia, Azerbaijan, Bangladesh, Belarus, Belize, Benin, Bhutan, Bolivia, Bosnia, Botswana, Brazil, Bulgaria, BurkinaFaso, Burundi, Cambodia, Cameroon, CaboVerde, Central African, Republic, Chad, China, Colombia, Comoros, Congo, CongoDemocratic public, CostaRica, Coted'Ivoire, Cuba, Djibouti, Dominican, Republic, Ecuador, Egypt, ElSalvador, Eritrea, Ethiopia, Fiji, Gabon, Gambia, Ghana, Grenada, Guatemala, Guinea, Guinea Bissau, Guyana, Haiti, Honduras, India, Indonesia, Iran, raq, Jamaica, Jordan, Kazakhstan, Kenya, Kiribati, Kyrgyzstan, Lao, Lebanon, Lesotho, Liberia, Libyan, Macedonia, Madagascar, Malawi, Malaysia, Maldives, Mali, Mauritania, Mauritius, Mexico, Moldova, Republic of Mongolia, Morocco, Mozambique, Myanmar, Namibia, Nepal, Nicaragua, Niger, Nigeria, Pakistan, Panama, Papua New Guinea, Paraguay, Peru Philippines, Romania, Rwanda, Saint Lucia Saint Vincent and the Grenadines Samoa Sao Tome and Principe Senegal, Serbia and Montenegro, Sierra Leone, Solomon Islands, South Africa, Sri Lanka, Sudan, Suriname, Swaziland, Syrian, Tajikistan, Tanzania, Thailand, Timorleste, Togo, Tonga, Tunisia, Turkey, Turkmenistan, Uganda, Ukraine, Uzbekistan, Vanuatu Viet Nam Yemen Zambia.
